# Identification of COVID-19 can be quicker through artificial intelligence framework using a mobile phone–based survey when cities and towns are under quarantine

**DOI:** 10.1017/ice.2020.61

**Published:** 2020-03-04

**Authors:** Arni S. R. Srinivasa Rao, Jose A. Vazquez

**Affiliations:** 1 Division of Health Economics and Modeling, Department of Population Health Sciences, Medical College of Georgia, Augusta University, Augusta, Georgia; 2 Laboratory for Theory and Mathematical Modeling, Division of Infectious Diseases, Department of Medicine, Medical College of Georgia, Augusta, Georgia; 3 Department of Mathematics, Augusta University, Augusta, Georgia; 4 Division of Infectious Diseases, Department of Medicine, Medical College of Georgia, Augusta University, Augusta, Georgia

## Abstract

We propose the use of a machine learning algorithm to improve possible COVID-19 case identification more quickly using a mobile phone–based web survey. This method could reduce the spread of the virus in susceptible populations under quarantine.

Emerging and novel pathogens are a significant problem for global public health. This is especially true for viral diseases that are easily and readily transmissible and have asymptomatic infectivity periods. The novel coronavirus (SARS-CoV-2) described in December 2019 (COVID-19) has resulted in major quarantines to prevent further spread, including major cities, villages, and public areas throughout China and across the globe.^1–3^ As of February 25, 2020, the World Health Organization’s situational data indicate ~77,780 confirmed cases in 25 countries, including 2,666 deaths due to COVID-19.^4^ Most deaths reported so far have been in China.^5^ The Centers for Disease Control and Prevention (CDC) and the World Health Organization have issued interim guidelines to protect the population and to attempt to prevent the further spread of the SARS-CoV-2 virus from infected individuals.^6^ Cities and villages throughout China are unable to accommodate such large numbers of infected individuals while maintaining the quarantine, and several new hospitals have been built to manage the infected individuals.^7^ It is imperative that we evaluate novel models to attempt to control the rapidly spreading SARS-CoV-2.^8^ Technology can assist in faster identification of possible cases to yield more timely interventions.

To reduce the time needed to identify a person under investigation (PUI) for COVID-19 and their rapid isolation, we propose to collect a basic travel history along with the more common signs and symptoms using a mobile phone–based online survey. Such data can be used in the preliminary screening and early identification of possible COVID-19 cases. Thousands of data points can be processed through an artificial intelligence (AI) framework that can evaluate individuals and stratify them into no risk, minimal risk, moderate risk, and high risk groups. The high-risk cases identified can then be quarantined earlier, thus decreasing the chance of spreading the virus (Table 1).

**Table 1.** Steps involved in the collection of data through a mobile phone-based survey

Step 1: Record the location details of the house/apartment from where the respondent uses a phone-based web survey/or the respondent’s usual place of stay.
Step 2: Record demographic information like gender (G) (1-male, 2-female, 3-others), age (A), race (R) (1-white, 2-black, 3-Hispanics, 4-Others)
Step 3: Have you traveled to (or living in) any of the COVID-19 affected areas/countries in the last 14 days? (Yes=1/No=0)
Step 4: Have you had any close contact with a person who is known to have COVID-19 during the last 14 days? (Yes=1/No=0)
Step 5: Record the presence or absence of signs and symptoms listed below and the duration of each of the signs and symptoms if yes to any of the signs and symptoms. A) fever (Yes=1/No=0), if yes, then the duration in days ---- B) cough (Yes=1/No=0), if yes, then the duration in days ---- C) shortness of breath (Yes=1/No=0), if yes, then the duration in days ---- D) myalgia or fatigue (Yes=1/No=0), if yes, then the duration in days ---- E) sputum production (Yes=1/No=0), if yes, then the duration in days ---- F) headache (Yes=1/No=0), if yes, then the duration in days ---- G) diarrhea (Yes=1/No=0), if yes, then the duration in days ---- H) pneumonia in both lungs (Yes=1/No=0), if yes, then the duration in days ----
Step 6: Enter the details of steps 1-5 above for any dependents or other individuals who live in the same location and do not have access to web-based survey.

Appendix 1 (online) lists the details of the steps involved in collecting data from all respondents independent of whether or not they think they are infected. The AI algorithm described in Appendix 2 (online) can identify possible cases and send an alert to the nearest health clinic as well as to the respondent for an immediate health visit. We call this an “alert for health check recommendation for COVID-19.” If the respondent is unable to commute to the health center, the health department can send an alert to a mobile health unit to conduct a door-to-door assessment and even test for the virus. If a respondent does not have an immediate risk of symptoms or signs related to the viral infection, then an AI-based health alert cab be sent to the respondent to notify them that there is no current risk of COVID-19. Figure 1 summarizes the outcomes of data collection and identification of possible cases.

**Fig. 1.**
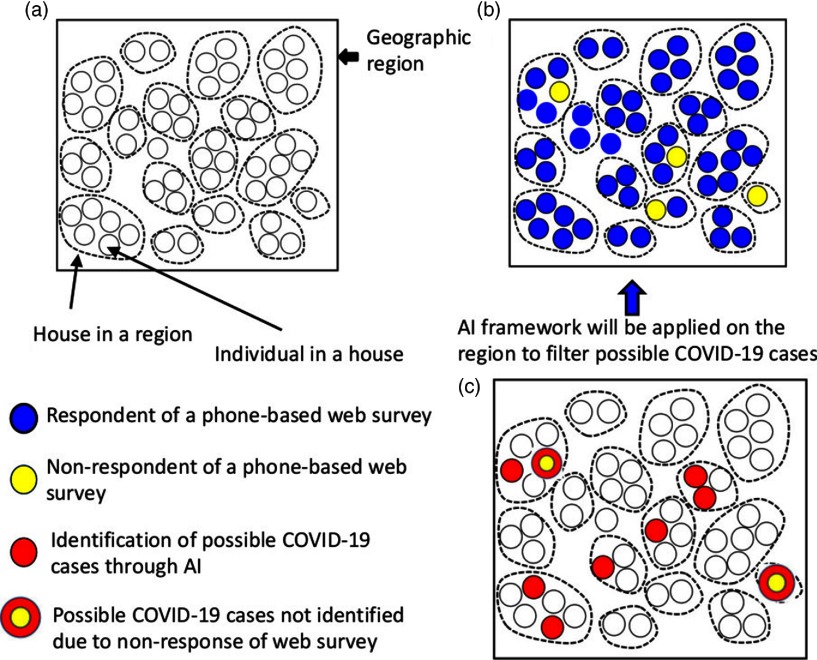
Conceptual framework of data collection and possible COVID-19 identification. (a) A geographical region (eg, a city, county, town, or village) with households in it. (b) Respondents and nonrespondents of a mobile phone–based web survey. (c) Possible identified cases of COVID-19 among the survey respondents and possible cases of COVID-19 among nonrespondents of the survey.

**Fig. 2.**
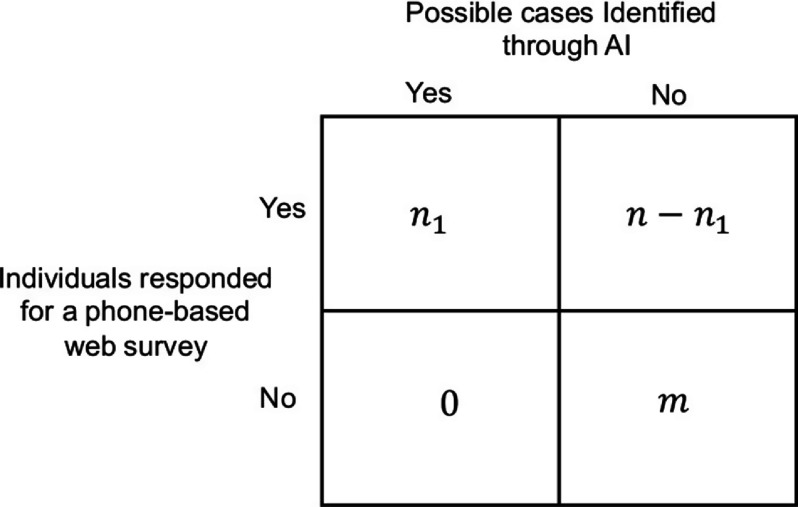
Number of possible cases identified through artificial intelligence (AI) framework versus the number of individuals who responded to a mobile phone–based web survey.

The signs and symptoms data recorded in step 5 of the algorithm are collected prior to Health Check Recommended for Coronavirus (HCRC) alerts or Health Check Recommended for Coronavirus (MHCRC) alerts (for possible identification and assessment) and No Health Check Recommended for Coronavirus (NCRC) alerts (for nonidentified respondents). These procedures are explained in steps 3 and 4 in Appendix 2. The extended analysis we propose can help determine any association among sociodemographic variables and the signs and symptoms, such as fever and lower respiratory infection including cough and shortness of breath, in individuals with and without possible infection. A 2 x 2 table of number of COVID-19 cases identified through AI and the number of people responded to a mobile survey is described in Figure 2.

Applications of AI and deep learning can be useful tools in assisting diagnoses and decision making in treatment.^10,11^ Several studies have promoted disease detection through AI models.^12–15^ The use of mobile phones^16–19^ and web-based portals^20,21^ have been tested successfully in health-related data collection. In addition, our proposed algorithm can be easily extended to identify individuals who might have any mild symptoms and signs. However, such techniques must be applied in a timely way for relevant and rapid results. Apart from cost-effectiveness, our proposed modeling method could greatly assist in identifying and controlling COVID-19 in populations under quarantine due to the spread of SARS-CoV-2.

## Appendix 1. Steps Involved in Data Collection Through Mobile Phones

We have developed our data collection criteria based on the CDC’s Flowchart to Identify and Assess 2019 Novel Coronavirus,^9^ and we have added additional variables for the extended utility of our efforts in identifying infected and controlling the spread (see Table 1 in the text).

## Appendix 2. Algorithm

Let ***O*_1_**, ***O*_2_**, ***O*_3_**, ***O*_4_**, ***O*_5_** be the outputs recorded during the data collection steps 1 through 5 described in the Appendix 1. The 3 outputs within ***O_2_*** are given as

and 9 pairs of outputs within ***O_5_*** are given as

where the pair ***O_5i_***, ***D_5i_*** for ***i = A, B, …I*** represents the respondent’s response regarding the presence or absence of ***i^th^*** sign and symptom (***O_5i_***) and duration of corresponding sign and symptom (***D_5i_***)

(1) If the set of identifiers, ***I*_1_**, for

is equal to one of the elements of the set ***C*_1_**, for

for a respondent, then, send HCRC or MHCRC. If ***I*_1_** is not equal to any of the elements of the set ***C*_1_** then proceed to test criteria (3).

(2) If the set of identifiers, ***I*_2_**, for

is equal to one of the elements of the set ***C*_1_**, then send HCRC or MHCRC to that respondent, else proceed to the test criteria (4).

(3) If ***I*_1_** is equal to one of the elements of the set ***C*_2_**, for

then the respondent will be sent an NCRC alert.

(4) If ***I*_2_** is equal to one of the elements of the set ***C*_2_**, then the respondent will be sent an NCRC alert.

A comparison of test criteria results of (3) and (4) with their corresponding geographic and sociodemographic details will yield further investigations of signs and symptoms based on whether or not an individual in the survey has traveled to coronavirus-affected areas or has had contact with any person who is known to have COVID-19. Here, we focus only on the identification of cases; further analysis techniques are beyond our scope. However, our approach is flexible enough to capture various other associations within the populations.

## Appendix 3. Further Computations on the Data Collected

Suppose ***n*** and ***m*** are individuals in a region who have responded and not responded, respectively, for a mobile phone–based online survey. Responses are randomly associated and not depended on the sickness due to the virus. The pair

yields the proportions of those who have responded and not responded in that region. Notably, we can compute  because the value ***m*** is known to us in that region. Here, ***n*_1_** of ***n*** are possible cases identified through our algorithm, and ***m*_1_** of ***m*** are possible cases of the virus that were not identified by the algorithm because ***m*** individuals never responded to the survey. Because ***n*** and ***m*** are known to us, one of the following relations will hold:

(A2.1)

Thus, we will see which of the relations listed in (A2.1) is true. When ***n***>m, one of the following relations will hold:

(A2.2)

However, we will never know which of the relations in (A2.1) is true because ***m*_1_** were never identified by the algorithm. For example, suppose 2,000 individuals respond to the survey, and of these, 500 individuals do not respond to the survey and 400 are identified as possible cases by the algorithm. If there are 100 possible cases of virus (which we do not have a mechanism to count) among the 500 who never responded, then the relation

is true. Similarly, other relations of (A2.2) could arise when ***n>m*** Using a similar argument, we can verify that when other relations of (A2.1) are true, we are still unsure which of the relations in (A2.1) are true. The **2 × 2** contingency options are provided in Figure 2 (in the text) to visualize the data to be generated using the proposed method.

*Theorem:* Let there be ***N*** individuals in a region. The probability that ***n*_1_** cases identified through the AI framework given that there are ***n*** individuals responded to the survey is

*Proof:* Let ***N* = *n* + *m***, and let

be the collection of ***n*** individuals who responded,

be the collection of ***m*** individuals who did not responded. Suppose

is the collection of respondents who are identified as possible cases. Here ***U*** ∪ ***V*** can be considered the region shown in (a), ***U*** shown in (b) and ***U*_1_** in (c) shown in Figure 1 (in the text).

Suppose we define 2 events ***E*_1_** and ***U*** using the sets ***U, V*** and ***U*_1_** as follows:

***E*_1_**: ***n*_1_** of ***n*** responded cases are identified through the algorithm

***E*** : ***n*** of ***N*** have responded to the survey.

The conditional probability of the event ***E*_1_** given the event ***E***, say, ***P*(E_1_/E)** is computed as follows:

▪
